# Pomegranate (*Punica granatum* L.) Extract and Its Anthocyanin and Copigment Fractions—Free Radical Scavenging Activity and Influence on Cellular Oxidative Stress

**DOI:** 10.3390/foods9111617

**Published:** 2020-11-06

**Authors:** Tina Kostka, Johanna Josefine Ostberg-Potthoff, Karlis Briviba, Seiichi Matsugo, Peter Winterhalter, Tuba Esatbeyoglu

**Affiliations:** 1 Institute of Food Science and Human Nutrition, Gottfried Wilhelm Leibniz University of Hannover, Am Kleinen Felde 30, 30167 Hannover, Germany; kostka@lw.uni-hannover.de; 2 Institute of Food Chemistry, Technische Universität Braunschweig, Schleinitzstrasse 20, 38106 Braunschweig, Germany; j.ostberg@tu-braunschweig.de (J.J.O.-P.); p.winterhalter@tu-braunschweig.de (P.W.); 3 Department of Physiology and Biochemistry of Nutrition, Max Rubner-Institut, Federal Research Institute of Nutrition and Food, Haid-und-Neu-Str.9, 76131 Karlsruhe, Germany; karlis.briviba@mri.bund.de; 4 School of Natural System, College of Science and Engineering, Kanazawa University, Kakuma-Machi, Kanazawa 920-1192, Japan; matsugoh@staff.kanazawa-u.ac.jp; 5 Department of Safety and Quality of Fruit and Vegetables, Max Rubner-Institut, Federal Research Institute of Nutrition and Food, Haid-und-Neu-Str.9, 76131 Karlsruhe, Germany

**Keywords:** pomegranate, HPLC, adsorptive membrane chromatography, electron spin resonance spectroscopy, cell culture, HepG2, antioxidant

## Abstract

Secondary plant metabolites, e.g., polyphenols, are widely known as health-improving compounds that occur in natural functional foods such as pomegranates. While extracts generated from these fruits inhibit oxidative stress, the allocation of these effects to the different subgroups of substances, e.g., anthocyanins, “copigments” (polyphenols without anthocyanins), or polymeric compounds, is still unknown. Therefore, in the present study, polyphenols from pomegranate juice were extracted and separated into an anthocyanin and copigment fraction using adsorptive membrane chromatography. Phenolic compounds were determined by high performance liquid chromatography with photodiode array (HPLC–PDA) detection and HPLC-PDA electrospray ionization tandem mass spectrometry (HPLC–PDA–ESI–MS/MS), while the free radical scavenging activity of the pomegranate XAD‑7 extract and its fractions was evaluated by the Trolox equivalent antioxidant capacity (TEAC) assay and electron spin resonance (ESR) spectroscopy. Compared to juice, the total phenolic content and free radical scavenging potential was significantly higher in the pomegranate XAD-7 extract and its fractions. In comparison to the anthocyanin and copigment fraction, pomegranate XAD-7 extract showed the highest radical scavenging activity against galvinoxyl and DPPH radicals. Moreover, the enriched XAD-7 extract and its fractions were able to protect human hepatocellular HepG2 cells against oxidative stress induced by hydrogen peroxide. Overall, these results indicated that anthocyanins and copigments act together in reducing oxidative stress.

## 1. Introduction

Over recent years, the consumption of so-called “superfoods” has become a new food trend as part of healthier and more conscious nutrition. Although there is no clear definition or food label for the term “superfood” [1], it generally comprises foods with specific health-improving properties, such as the reduction of reactive oxygen species (ROS) or anti-proliferative activities for cancer prevention [2,3].

One of the most popular superfoods is the subtropical fruit pomegranate (*Punica granatum* L.), which belongs to the family Punicaceae. Pomegranates are native to Iran and are nowadays cultivated in North Africa as well as in North and South America [4,5]. Worldwide, around 300,000 ha of pomegranate is cultivated [6]. Similar to red wine, tea, and fruit juices, pomegranate contains a high content of soluble phenolic compounds, which are well-known antioxidants [3,7,8,9], occuring in the seeds, peel, and fruit [10]. The tightly packed juicy arils of pomegranate are a rich source of punicalagins, ellagic acid, and flavonoids, such as anthocyanins [5,11]. While these secondary metabolites are associated with antioxidant activity, inhibition of DNA damage, and suppression of cancer cell growth [7,12,13,14,15], their bioavailability in the small intestine is low [16,17]. For instance, the consumption of 300 g of red fruits led to non-detectable contents of anthocyanins and ellagic acids in human plasma samples [17]. Thus, a high amount of fruit needs to be consumed in order to reach sufficient concentrations of natural antioxidants. Despite of their low bioavailability, phenolic compounds represent effective substances for usage as nutraceuticals and/or food additives by generation of bioactive-enriched fruit extracts. Pharma and industrial companies are increasingly focusing on the generation of such extracts enriched with bioactive compounds. Moreover, by recycling of agricultural wastes such as pomegranate peels, whose extracts have shown high radical scavenging activities, food wastes will be reduced and health-improving resources will be obtained [18].

In several in vitro and in vivo studies, the health-promoting benefits of pomegranate extracts (PE) were analyzed. PE showed a significant antioxidant potential, decreased the intracellular ROS formation, and could lead to G2/M cell cycle arrest followed by apoptosis [14,19,20]. Additionally, preventive effects against DNA damage and chromosome aberrations were detected [13,19,21]. In all of the aforementioned studies, the entire PE consisting of a complex mixture of polyphenols, tannins, and anthocyanins, was analyzed regarding its health-improving effects [5,20]. Seeram and coworkers [22] detected a significantly higher inhibition of cell proliferation induced by the entire polyphenol fraction compared to anthocyanins alone. Moreover, Gil et al. [7] analyzed the antioxidant activity before and after removing the anthocyanin fraction from pomegranate juice. The remaining phenolic compounds reached only 28% of the total activity, indicating the importance of anthocyanins for the antioxidant effects of pomegranate fruits. Owing to these substance-dependent properties, the attribution of the observed biological effects to the responsible substances is difficult and should be considered in further studies [22,23]. Such results would improve the selection of bioactive compounds, e.g., anthocyanins or ellagitannins, for producing more efficient nutraceuticals.

Therefore, the aim of the present study was to fractionate the phenolic constituents of pomegranates by adsorptive membrane chromatography into the polymeric and monomeric compounds, while the latter group was separated into an anthocyanin and copigment fraction, which consists of various phenolic compounds [24]. In order to detect substance specific differences and the most effective compounds concerning the bioactivity of the extract and the separated fractions, we examined their radical scavenging activity as well as the cellular-protective potential against oxidative stress.

## 2. Materials and Methods

### 2.1. Chemicals

Double deionized water was obtained by Nanopure resin (Werner, Leverkusen, Germany). Amberlite XAD-7, 2,2′-azino-bis-(3-ethylbenzothiazolin-6-sulfonic acid) (ABTS, 98%), ethanol (HPLC grade), catechin hydrate, and trypsin were purchased from Sigma-Aldrich (Steinheim, Germany). Acetonitrile (LC–MS and HPLC grade) was obtained from Honeywell (Seelze, Germany). Formic acid (LC–MS grade) and *n*-hexane (>95%) were obtained from Fisher Scientific (Geel, Germany). Formic acid (HPLC grade) was ordered from AppliChem GmbH (Darmstadt, Germany). Acetic acid (HPLC grade) and hydrogen peroxide (H_2_O_2_, 30%, stabilized) were obtained from VWR International GmbH (Leuven, Germany). Folin–Ciocalteu reagent, dimethyl sulfoxide, p.a. (pro analysis), and Muse Oxidative Stress Kit were purchased from Merck KGaA (Darmstadt, Germany). Gallic acid monohydrate and 6-hydroxy-2,5,7,8-tetramethylchromane-2-carboxylic acid (Trolox, ≥98% purum) were acquired from Fluka (Buchs, Switzerland). Potassiumperoxodisulfate (K_2_S_2_O_8_) was obtained from Riedel-de-Haën (Seelze, Germany). Sodium carbonate (≥99% p.a.), sodium chloride (≥99% p.a.), and sodium hydroxide (≥99% p.a.) were ordered from Carl Roth GmbH + Co. KG (Karlsruhe, Germany). Galvinoxyl radical and DPPH radical were purchased from Tokyo Chemical Industry (TCI; Tokyo, Japan). Triton X was obtained from Serva Electrophoresis (Heidelberg, Germany). Cell Proliferation Kit I (MTT, 3-(4,5-Dimethylthiazol-2-yl)-2,5-diphenyltetrazolium bromide) was ordered from Roche (Mannheim, Germany). Dulbecco’s phosphate-buffered saline (DPBS, without Ca and Mg) as well as Hanks’ balanced salt solution (HBSS, without phenol red, with Ca, Mg, and 0.35 g/L NaHCO_3_) were purchased from PAN Biotech (Aidenbach, Germany).

### 2.2. Cell Culture

Human hepatoma cell line HepG2 (DSMZ-No: ACC-180) was purchased from the German Collection of Microorganisms and Cell Cultures (DSMZ, Braunschweig, Germany). The cells were cultured in RPMI (Roswell Park Memorial Institute) 1640 medium enriched with L-glutamine, 2.0 g/L NaHCO_3_ (PAN Biotech, Aidenbach, Germany), 10% (*v*/*v*) fetal bovine serum (FBS; Fisher Scientific, Schwerte, Germany), 100 IU/mL penicillin, and 100 μg/mL streptomycin (Life Technologies, Darmstadt, Germany). For optimal cell cultivation, we kept the cells at 37 °C under a humidified atmosphere of 95% air and 5% CO_2_ by replacing the medium every 2–3 days. Once a week, the cells were passaged using the cell passages 20–25 for the following in vitro experiments.

### 2.3. Pomegranate (Punica granatum L.) Sample

A commercially available pomegranate juice NFC (not from concentrate; *Punica granatum* L.) was kindly provided from Haus Rabenhorst O. Lauffs GmbH and Co. KG (Unkel, Germany). In three independent experiments, we generated pomegranate extracts from the juice.

### 2.4. Preparation of a Phenolic Fruit Extract from Pomegranate Juice by Adsorption on an Amberlite XAD-7 Resin

**Figure 1:**
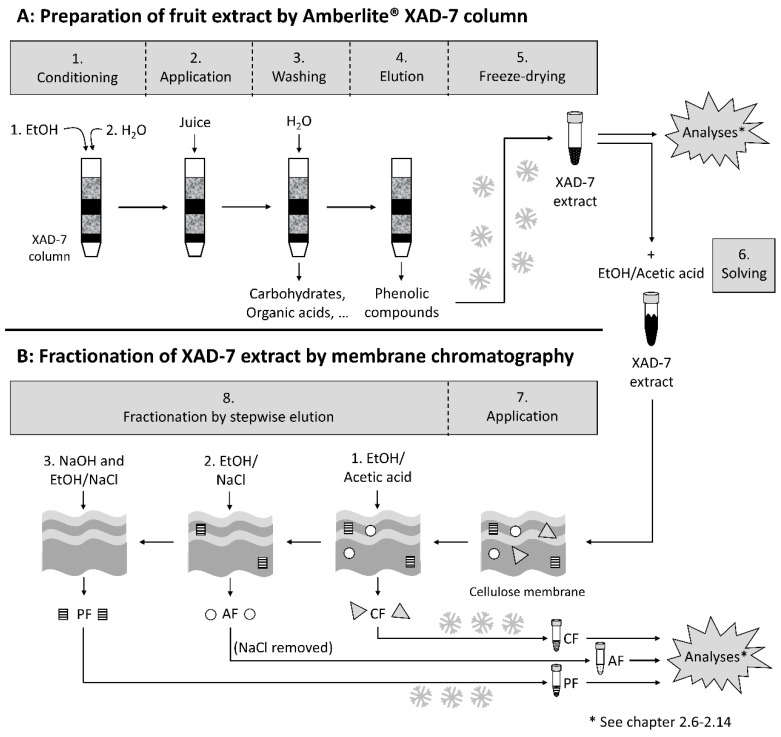
Schematic overview of the preparation of the pomegranate XAD-7 extract (**A**) and its separated fractions (**B**; PF: polymeric fraction; AF: anthocyanin fraction; CF: copigment fraction).

A scheme comprising the following methodology is shown in Figure 1A. Pomegranate juice NFC (1.49 L) was applied onto an Amberlite XAD-7 column (100 × 7 cm), which had been conditioned with 2 L of ethanol and then with 2 L of water. The XAD-7 column was washed with water in order to eliminate carbohydrates, proteins, organic acids, and minerals. Retained phenolic compounds were eluted with ethanol/acetic acid (19:1; *v*/*v*). Solvents were evaporated under reduced pressure at <40 °C, and the XAD-7 extract was freeze-dried. About 6 g pomegranate XAD-7 extract was obtained.

### 2.5. Fractionation by Adsorptive Membrane Chromatography

With reference to Juadjur and Winterhalter [24] and Ostberg-Potthoff et al. [25], anthocyanins (λ = 520 nm) were separated from other non-colored phenolic compounds, so-called copigments (λ = 280 nm), on a cellulose membrane adsorber (type Sartobind S IEX 150 mL) from Sartorius (Göttingen, Germany; Figure 1B). Briefly, pomegranate XAD-7 extract (4.96 g) was dissolved in 1 L ethanol/acetic acid (19:1; *v*/*v*) and applied onto the cellulose membrane. First, copigments were eluted with 1 L ethanol/acetic acid (19:1; *v*/*v*). Afterwards, the retained anthocyanins were eluted with 1 L of a mixture of an aqueous NaCl (1 N) solution and ethanol (1:1; *v*/*v*). Both fractions were concentrated in vacuo and the residue was freeze-dried. NaCl from the anthocyanin fraction was removed by Amberlite XAD-7, as described in Section 2.4. The anthocyanin fraction was eluted with ethanol/acetic acid (19:1; *v*/*v*), and the solvents were evaporated under reduced pressure at <40 °C and freeze-dried. To remove the polymers from the membrane, the membrane was regenerated with 1 N NaOH and the polymers were eluted with a mixture of NaCl (0.1 N) and ethanol (2:8; *v*/*v*). For detailed information, see Ostberg-Potthoff et al. [25].

### 2.6. Quantification of the Total Phenolic Content (TPC) Using the Folin–Ciocalteu Assay

The TPC was determined using the Folin–Ciocalteu assay according to Singleton and Rossi [26] with slight modifications. Briefly, 200 µL of a test sample (1 mg/mL), a standard, and a blank sample (water) were each mixed with 1 mL diluted Folin–Ciocalteu reagent (1:10; *v*/*v*). After 7 min, 800 µL 7.5% Na_2_CO_3_ solution was added, followed by an incubation for 2 h at room temperature. Afterwards, the absorbance was measured at λ = 760 nm using a V-750 UV-visible/NIR (Near-infrared) spectrophotometer (Jasco, Gross-Umstadt, Germany). The TPC was calculated by subtraction of the value of the blank sample, followed by quantification with a calibration curve established using 11–66 mg/L gallic acid. The results were expressed as grams of gallic acid equivalents per 100 g extract (g GAE/100 g). The measurements were repeated three times.

### 2.7. Quantification of Polyphenols by UHPLC–PDA

UHPLC–PDA (Ultra high performance liquid chromatography with photodiode array) quantification of anthocyanins, phenolic acids, and flavonols were performed on a Zorbax Eclipse Plus C18 (1.8 µ, 50 mm × 2.1 mm) column (Agilent, Waldbronn, Germany) using a 1290 Infinity II UHPLC system (Agilent, Waldbronn, Germany). The quantification was carried out with solvent systems A (5% formic acid) and B (acetonitrile) at a flow rate of 0.8 mL/min and 30 °C and the following gradient: 0 min 0% B, 12 min 5% B, 17 min 5% B, 23 min 7% B, 25 min 10% B, 27 min 15% B, 28 min 30% B, 29.5 min 95% B, 31 min 95% B, 33 min 0% B, 35 min 0% B. Quantification of tannins was performed on a Synergi Hydro-RP (4 µ, 250 × 4.6 mm) column (Phenomenex, Aschaffenburg, Germany). Solvent system A was Nanopure and solvent system B was 0.5% acetic acid/methanol (10:90; *v*/*v*) at a flow rate of 0.6 mL/min and 30 °C. The gradient was as follows: 0 min, 0% B; 1 min, 10% B; 25 min, 15% B; 32 min, 20% B; 35 min, 25% B; 60 min, 50% B; 63 min, 100% B; 63 min, 100% B; 66 min, 100% B; 68 min, 0% B; 72 min, 0% B. Anthocyanins were detected at λ = 520 nm, phenolic acids at λ = 280 nm or 320 nm, flavonols at λ = 360 nm, and tannins at λ = 320 nm. The quantification was carried out in triplicate, employing cyanidin-3-*O*-glucoside (0.004–1 mg/mL, LOQ (limit of quantification) 0.13 mg/mL, LOD (limit of detection) 0.001 mg/mL) as an external standard for anthocyanins, chlorogenic acid (5-CQA, 0.003–1 mg/mL, LOQ 0.04 mg/mL, LOD 0.001 mg/mL) as a standard for phenolic acids, quercetin-3-*O*-glucoside (0.005–1.1 mg/mL, LOQ 0.14 mg/mL, LOD 0.004 mg/mL) as standard for flavonols, and punicalin (0.005–1 mg/mL, LOQ 0.25 mg/mL, LOD 0.005 mg/mL) as standard for tannins.

### 2.8. Quantification of Polymeric Compounds by HPLC–PDA

HPLC–PDA quantification of polymeric compounds were performed on a Primesep B2, 5 µ, 150 mm × 2.1 mm column (SIELC, Wheeling, WV, USA) using a PU-2080 Plus Intelligent HPLC pump, a DG-2080-50 3-line Degasser, a LG-2080-02 Ternary Gradient Unit, an AS-2057 Plus Intelligent Sampler, a CO-2067 Plus Intelligent Column Oven, a MD-2010 Plus Multiwavelength Detector, and a LC-NetII/ADC Transmitter (Jasco, Gross-Umstadt, Germany). The quantification was carried out with solvent systems A (5% aqueous formic acid) and B (acetonitrile) at a flow rate of 0.2 and 0.4 mL/min and 40 °C using the following gradient: 0 min, 0% B; 20 min, 0% B; 60 min, 15% B; 65 min, 50% B; 70 min, 50% B; 75 min, 100% B; 80 min, 100% B; 85 min, 0% B; 100 min, 0% B. The quantification was carried out with catechin as standard (0.01–1 mg/mL, LOQ 0.09 mg/mL, LOD 0.002 mg/mL) at λ = 280 nm.

### 2.9. Polyphenol Identification by HPLC Photodiode Array Electrospray Ionization Tandem Mass Spectrometry (HPLC–PDA–ESI–MS/MS)

HPLC–PDA–ESI–MS/MS analyses were conducted according to Ostberg-Potthoff et al. [25]. Therefore, an HPLC system (1100/1200 series, Agilent, Waldbronn, Germany) coupled to an HCT (high capacity spherical trap) Ultra Ion Trap mass spectrometer (Bruker Daltonics, Bremen, Germany) with an electrospray ionization source (ESI) was applied.

### 2.10. Determination of the Radical Scavenging Capacity by the Trolox Equivalent Antioxidant Capacity (TEAC) Assay

The pomegranate XAD-7 extract and its anthocyanin, copigment, and polymeric fractions were analyzed regarding their radical scavenging capacity by the TEAC assay. One milliliter of an ABTS solution (7 mM ABTS and 2.5 mM of K_2_S_2_O_8_ dissolved in ultrapure water overnight and diluted with ethanol) was mixed with 20 µL of the samples (10 mg/10 mL), a standard, or a blank solution, and incubated for 12 min at room temperature. The radical scavenging effect, which was measured at λ = 734 nm using a V-750 UV-visible/NIR spectrophotometer (Jasco, Gross-Umstadt, Germany), was quantified by a Trolox standard curve prepared in ethanol, while all samples were analyzed in triplicate. The results were expressed as millimole of Trolox equivalents per 100 g sample (mmol TE/100 g).

### 2.11. Determination of the Free Radical Scavenging Activities by ESR Spectroscopy

Electron spin resonance (ESR) spectroscopy measurements were performed according to Esatbeyoglu et al. [27] and Noda et al. [28] by a JEOL JES-FR30 EX free radical monitor (Jeol Ltd., Akishima, Japan) with the following experimental conditions: microwave power of 4 mW, sweep width of 7.5 mT, sweep time of 2 min, modulation of 100 KHz, 0.32 mT, amplitude of 3.2 × 10^2^ (galvinoxyl radical), 5.0 × 10^2^ (DPPH radical), time constant of 0.3 s, accumulation of 1, and center field of 337.394 mT.

Test samples: High concentrations of all samples (5, 10, or 20 mg/mL) were dissolved in water/ethanol (9:1; *v*/*v*). This solution was further diluted with water/ethanol (8:2; *v*/*v*) to obtain working solutions with 1, 0.8, 0.6, 0.4, 0.2, 0.1, 0.05, and 0.01 mg/mL concentrations.

Experimental procedure for DPPH and galvinoxyl radicals:

DPPH radicals: At first, 10 µL of sample (10, 50, 100, 200, 400, 600, 800, 1000 µg/mL; final concentration of all samples were 1, 5, 10, 20, 40, 60, 80, and 100 µg/mL) were mixed with 30 µL of water/ethanol (8:2; *v*/*v*) solution, followed by adding 60 µL of ethanol solution containing 1.67 mM of DPPH (final concentration of DPPH was adjusted to 1 mM). Then, the solution was vortexed and centrifuged for about 10 s. After centrifugation, the mixed solution was taken up in a capillary tube (around 50 µL). The control experiment was carried out with 40 µL of water/ethanol (8:2; *v*/*v*) solution and ethanol solution containing 1.67 mM of DPPH.

Galvinoxyl radicals: Similar to DPPH, 10 µL of sample was mixed with 40 µL of water/ethanol (8:2; *v*/*v*) solution and 50 µL of ethanol solution containing 1 mM of galvinoxyl radical (final concentration of galvinoxyl radical was adjusted to 0.5 mM). After mixing of all components, the solution was vortexed and centrifuged for approximately 10 s, followed by transferring the mixture in a capillary tube (around 50 µL). The control experiment was carried out with 50 µL of water/ethanol (8:2; *v*/*v*) and ethanol containing 0.5 mM of galvinoxyl solution.

All experiments were performed in triplicate.

### 2.12. Preparation of XAD-7 Extract and Its Separated Fractions for Cell Culture Experiments

For cell treatment, the freeze-dried XAD-7 extract as well as the anthocyanin and copigment fraction, were dissolved in DMSO and sterile filtered (0.22 µm) to generate stock solutions (100 mg/mL). In the following in vitro assays, DMSO served as solvent control for all test samples.

### 2.13. Determination of the Cytotoxic Potential on HepG2 Cells in the MTT Viability Assay

At the beginning of the in vitro assays, we determined the cytotoxicity of the different fractions using the MTT viability assay. Briefly, 2 × 10^4^ HepG2 cells per well and 8 wells per test substance were seeded in 96-well plates (Greiner Bio-One, Frickenhausen, Germany). Approximately 24 h later, the medium was removed and replaced by the treatment media containing different concentrations of the test samples (0, 25, 50, and 100 µg/mL). According to the manufacturer’s instructions of the MTT assay, the cell treatment occurred for 24 h, whereas 0.1% DMSO served as solvent and 1% Triton X as positive control. For the cell viability measurement, we washed the cells using a phosphate-buffered saline (PBS), followed by an incubation with a 1:10 (*v*/*v*) dilution of the MTT reagent in medium for 4 h at 37 °C. Then, after metabolic MTT reduction, the samples were incubated overnight at room temperature with the solubilization solution. Finally, the cell viability was determined by measuring the absorbance at λ = 595 nm using a microplate absorbance reader (iMark, Bio-Rad Laboratories GmbH, Feldkirchen, Germany) with the included Biorad Microplate Manager Software 6.2 (Feldkirchen, Germany).

### 2.14. Quantitative Determination of ROS Reducing Activity

For quantification of antioxidant effects, we treated HepG2 cells with PE and its fractions, followed by an incubation with H_2_O_2_ as a ROS-inducer and analysis using the specific detection reagent of the Muse Oxidative Stress Kit (Merck, Darmstadt, Germany).

In more detail, 1 × 10^6^ cells per well were seeded with 3 mL medium in a 6-well plate (Greiner Bio-one, Frickenhausen, Germany) and cultured for 24 h at 37 °C and 5% CO_2_. Then, the cells were treated for 2 h with the pomegranate XAD-7 extract or its fractions (75 µg/mL pomegranate samples) using HBSS as a medium replacement. Alternatively, for detection of an antioxidant capacity, the cells were incubated for 1 h with the samples or 0.1% DMSO as solvent control, followed by a treatment with a non-cytotoxic concentration of H_2_O_2_ (100 µM; known from earlier studies), also diluted in HBSS, for another incubation time of 1 h at 37 °C. After removal of the treatment solutions, the cells were washed with PBS, trypsinized with 200 µL trypsin per well for 5 min at 37 °C, and transferred with 400 µL HBSS in a centrifugation tube (1.5 mL Eppendorf). The suspensions were centrifuged for 2 min at 1000 g and 4 °C using the centrifuge Type Micro Star 17R from VWR (Darmstadt, Germany). The supernatant was removed, and the pellet was re-suspended with 200 µL assay buffer of the Muse Oxidative Stress Kit. Finally, 10 µL of the cell suspension was mixed with 190 µL of a prepared Muse Oxidative Stress working solution, incubated for 30 min at 37 °C. The ROS generation was analyzed by the Muse Cell Analyzer (Merck, Darmstadt, Germany). For each sample, the percentage of ROS-positive and ROS-negative cells was calculated automatically.

### 2.15. Statistical Analysis

Statistical analysis was carried out using the software Prism (version 8.4.1; GraphPad, La Jolla, CA, USA). Data were analyzed for normality of distribution by Shapiro–Wilk test, followed by a one-way ANOVA including Tukey’s multiple comparison test for normally distributed data. Alternatively, the Kruskal–Wallis test followed by Dunn’s multiple comparison post-hoc analysis was performed for detecting significant differences within experimental data, showing no normal distribution. Differences were considered as significant when *p* < 0.05, while data are shown as means ± SD (Standard Deviation) of at least three independent experiments.

## 3. Results

### 3.1. Chemical Characterization of PE and Its Fractions Obtained by Membrane Chromatography

**Figure 2:**
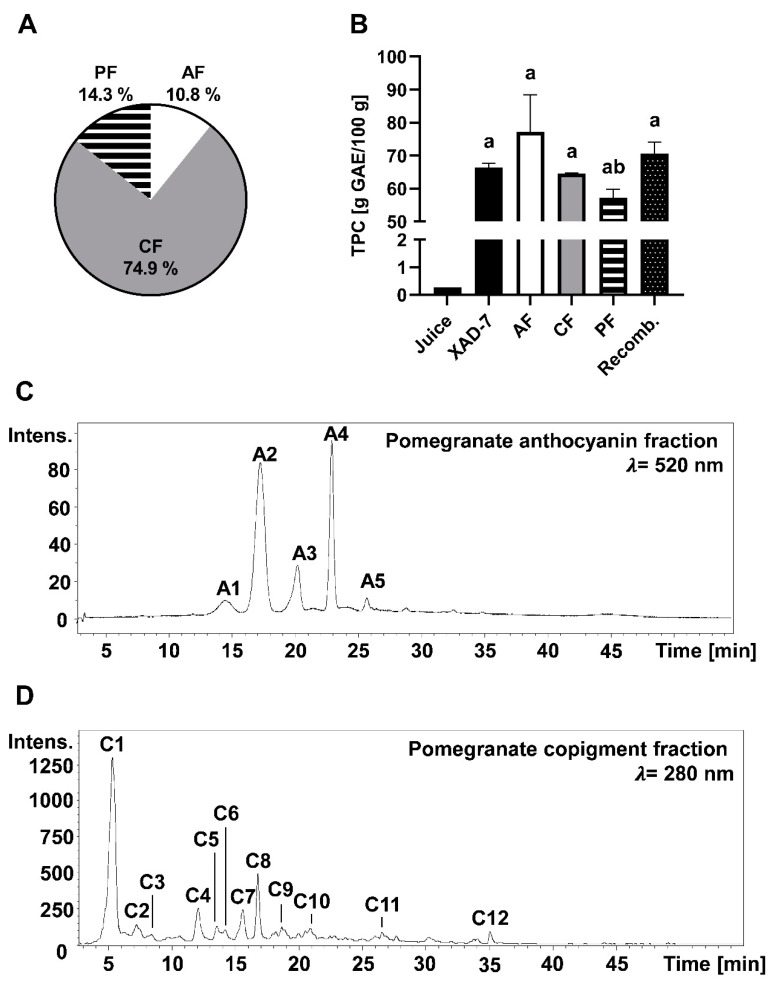
Chemical characterization of pomegranate XAD-7 extract and its separated fractions including the anthocyanin fraction (AF), the copigment fraction (CF), and the polymeric fraction (PF). Shown is the mean ± SD (standard deviation) of at least three independent experiments. (**A**) Composition of pomegranate XAD-7 extract. (**B**) Total phenolic content (TPC) of pomegranate juice, its XAD-7 extract, anthocyanin, copigment and polymeric fraction, and the recombined fractions (Recomb.). Statistical analysis was performed using one-way ANOVA followed by Tukey’s multiple comparison test. a: significantly different to juice with *p* < 0.05; b: significantly different to AF with *p* < 0.05. HPLC chromatogram of the anthocyanin fraction (**C**) and copigment fraction (**D**). Peak identification is given in Table 1. Intens.: Signal intensity.

About 6.16 g of a phenolic-enriched pomegranate (*Punica granatum* L.) XAD-7 extract was obtained from 1.56 kg pomegranate juice NFC. The obtained pomegranate XAD-7 extract was composed of 2.06 g/100 g (±0.074 g/100 g) anthocyanins, 53.4 g/100 g (±3.20 g/100 g) copigments (mainly tannins), and 8.47 g/100 g polymeric compounds. After separation of the different fractions by a cellulose membrane adsorber, a relative content of 11% anthocyanins, 14% polymeric compounds, and 75% copigments were obtained from the pomegranate XAD-7 extract (Figure 2A). The different fractions, as well as the pomegranate XAD-7 extract and the pomegranate juice, were analyzed for the TPC using the Folin–Ciocalteu assay. TPC of the juice was lower (0.27 g GAE/100 g) compared to the enriched pomegranate XAD-7 extract and its fractions, for which TPC content ranged from 57 to 77 g GAE/100 g (Figure 2B). After recombining the monomeric and polymeric fractions according to the analyzed composition, we achieved the TPC content of the pomegranate XAD-7 extract.

Compounds of pomegranate anthocyanin and copigment fraction were identified by HPLC–ESI–MS/MS analyses. In pomegranate, cyanidin, delphinidin, and pelargonidin derivates were detected (Table 1). Cyanidin-3,5-diglucoside (*m*/*z* 611), delphinidin-3-glucoside (*m*/*z* 465), and cyanidin-3-glucoside (*m*/*z* 449) are the main pigments. Major copigments in pomegranate are the ellagtannins, with punicalin (*m*/*z* 781) being the main compound. HPLC chromatograms of the anthocyanin and copigment fractions at λ = 520 nm and λ = 280 are shown in Figure 2C,D.

### 3.2. Detection of Free Radical Scavenging Activity

**Figure 3:**
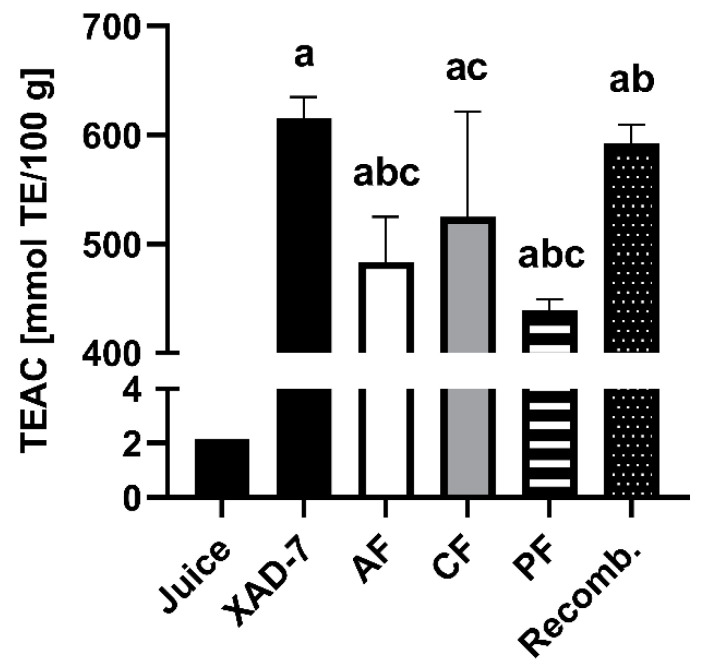
Total antioxidant capacity shown as Trolox equivalent (measured by Trolox equivalent antioxidant capacity (TEAC) assay) of pomegranate juice; XAD-7 extract; its anthocyanin (AF), copigment (CF), and polymeric fractions (PF); and the recombined extract (Recomb.). Results are presented as mean ± SD of three independent experiments. For statistical analysis, one-way ANOVA followed by Tukey’s multiple comparison test was performed. a: significantly different to juice with *p* < 0.05; b: significantly different to XAD-7 with *p* < 0.05; c: significantly different to Recomb. with *p* < 0.05.

**Figure 4:**
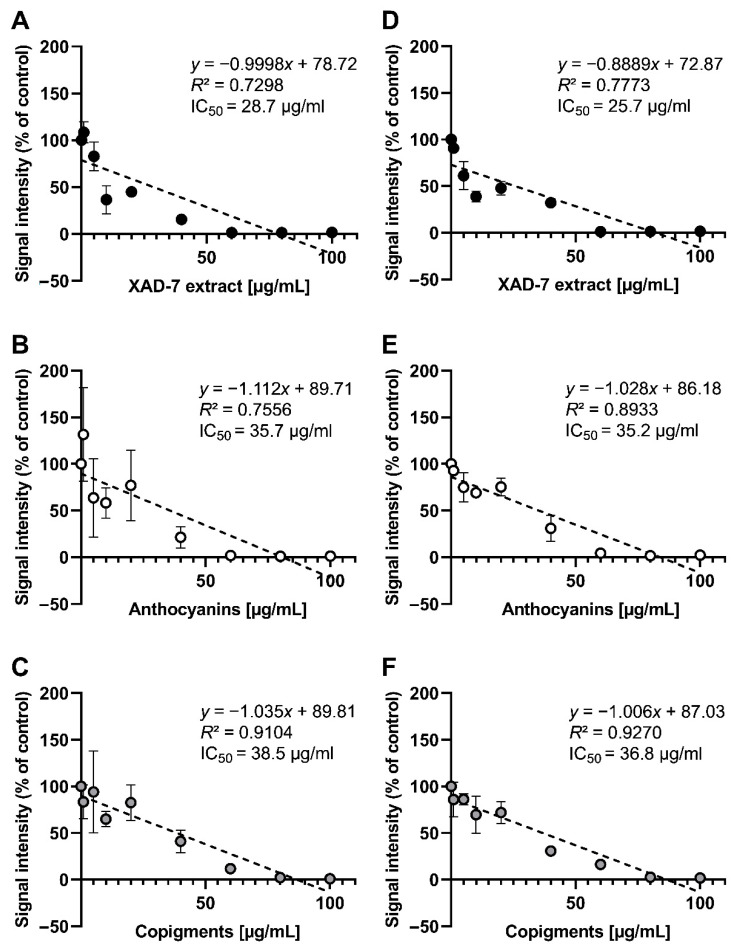
Total antioxidant galvinoxyl radical (**A**–**C**) and DPPH radical (**D**–**F**) scavenging activity of pomegranate XAD-7 extract and its fractions determined by electron spin resonance (ESR) spectroscopy. Shown is the mean ± SD, the linear equation, as well as the calculated *R*^2^ and IC_50_ values of three independent experiments.

The radical scavenging capacity was evaluated for the pomegranate XAD-7 extract and its fractions, as well as the recombined extract using spectrophotometric assays, such as TEAC (scavenging of ABTS^+∙^-radicals; Figure 3) and the ESR method scavenging activity against galvinoxyl and DPPH radicals (Figure 4). The XAD-7 extract of pomegranate, determined by the TEAC assay, had the highest radical scavenging activity, equally shown for the recombined fraction, which was composed of the anthocyanin, copigment, and polymeric fraction obtained after membrane separation. The results of each separated fraction was lower, with a minimal TEAC value for the polymeric fraction with 439 mmol TE/100 g, compared to the pomegranate XAD-7 extracts (615 and 592 mmol TE/100 g). Additionally, free radical scavenging activities of pomegranate XAD-7 extract and its anthocyanin and copigment fraction were examined using ESR spectroscopy. According to the ESR results, all samples showed moderate scavenging activities against galvinoxyl and DPPH radicals (IC_50_ values). In the case of the radical scavenging activity of galvinoxyl radicals, the entire pomegranate XAD-7 extract (IC_50_ = 28.7 µg/mL) showed the strongest radical scavenging activity compared to pomegranate copigment (IC_50_ = 38.5 µg/mL) and anthocyanin fraction (IC_50_ = 35.7 µg/mL; Figure 4A–C). This observation was also found in the case of DPPH radical scavenging activities (Figure 4D–F). All analyzed samples exhibited scavenging activity against galvinoxyl and DPPH radicals in a dose-dependent manner.

### 3.3. Analyzing the Protection Potential against ROS Induced by H_2_O_2_

**Figure 5:**
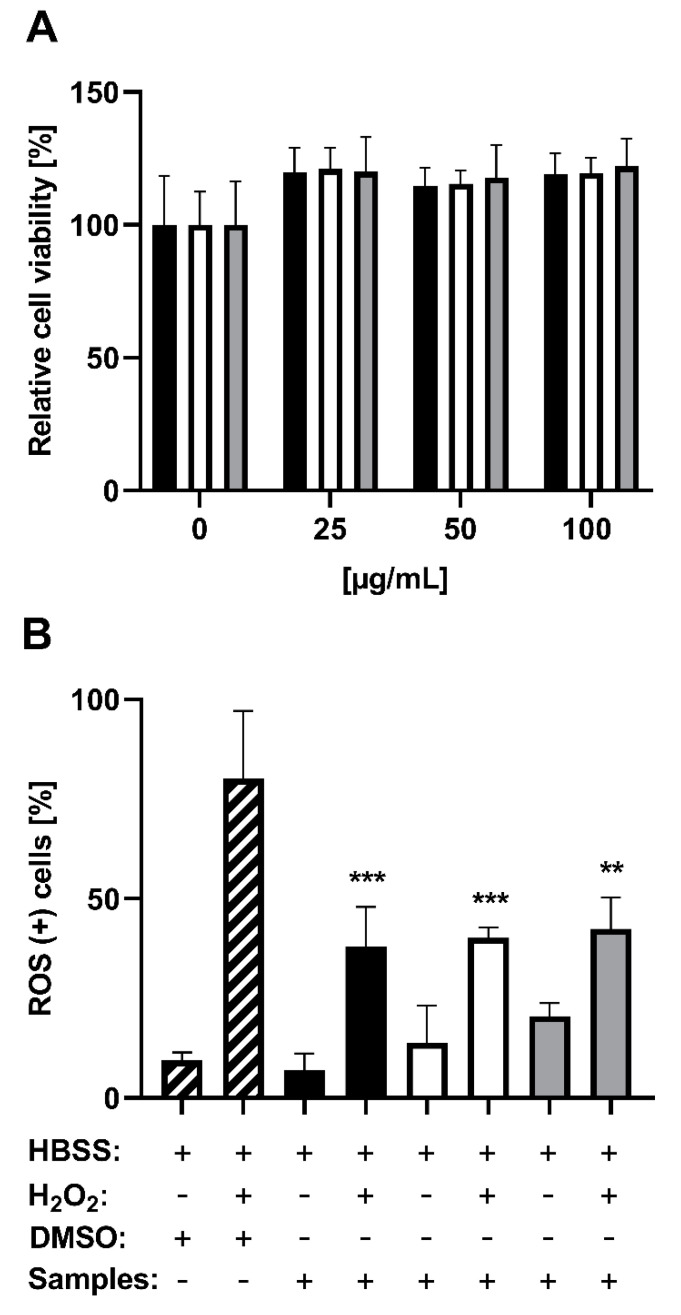
In vitro characterization of cytotoxic and reactive oxygen species (ROS)-reducing effects of pomegranate XAD-7 extract (black bars), the anthocyanin fraction (white bars), and the copigment fraction (grey bars) using HepG2 cells. Shown is the mean ± SD of three independent experiments. (**A**) MTT assay using 0.1% DMSO as solvent control (0 µg/mL). Statistical analysis was performed by the Kruskal–Wallis test followed by Dunn’s multiple comparison post-hoc analysis, showing no significant difference between all samples. (**B**) ROS-reducing effects of pomegranate samples (each 75 µg/mL) or 0.1% DMSO with or without incubation with H_2_O_2_ (100 µM)-all dissolved in HBSS. For significance detection, the samples with or without H_2_O_2_ post-treatment were compared to the H_2_O_2_-treated or non-treated sample-free controls. Statistical analysis was performed using the one-way ANOVA followed by Tukey’s multiple comparison test. ** *p* < 0.01, *** *p* < 0.001.

In addition to the radical scavenging activity, the potential of ROS reduction by PE as well as its anthocyanin and copigment fraction was determined in vitro using human hepatocellular carcinoma HepG2 cells. At first, to exclude cellular effects or mechanisms due to cytotoxicity, we analyzed all tested samples using the MTT viability assay. The results at concentrations of 25–100 µg/mL of the pomegranate XAD-7 extract and fractions showed no decrease, but a slight non-significant increase in cell viability (Figure 5A). With reference to the results of the radical scavenging activities, showing a full signal inhibition at concentrations of 60–80 µg/mL extract or fraction, we tested a similar concentration (75 µg/mL) in the cell culture experiments. For the examination of antioxidant activity, we counted ROS-positive HepG2 cells after pretreatment with the pomegranate samples, followed by incubation with or without H_2_O_2_. While no significant increase in ROS-positive cells by the extract and fractions themselves was detected, all pomegranate samples showed significant protective effects against H_2_O_2_-induced toxicity in HepG2 cells (Figure 5B). While H_2_O_2_ led to 80% ROS-positive cells, the pretreatment with pomegranate samples significantly reduced this value to 40%, with no significant difference between the used fractions.

## 4. Discussion

Compared to other red fruit extracts, e.g., aronia, elderberry, blueberry, red grape, and blackcurrant, the analyzed PE contained less anthocyanins, but high amounts of hydrolysable tannins as part of the copigment fraction [29]. Independent of the separation of the different fractions, the TPC was similar for all samples, except of the pomegranate juice, which showed a significantly lower TPC. Nevertheless, the reported TPC of 2700 mg/L pomegranate juice is comparable with data from the literature, ranging from 1680 to 5260 mg/L [3,7,30]. However, by generation of the XAD-7 extract, the TPC of pomegranate juice could be enriched, which may result in a higher antioxidant capacity, additionally to the fact that PE showed the highest TPC of the abovementioned fruit extracts [29]. After recombining all separated fractions, the TPC as well as the radical scavenging activity measured by TEAC assay of the recombined was totally recovered. These results highlight the high efficiency of the fractionation method, enabling a gentle separation of anthocyanins from copigments and polymeric compounds without loss of antioxidant activity.

The composition of the phenolic compounds was further separated and characterized. The results of the composition analyses were similar to the results of Fischer et al. [31], Di Stefano et al. [32], and Akhavan et al. [33], who identified cyanidin-3,5-diglucoside, delphinidin-3-glucoside, and cyanidin-3-glucoside as the compounds with the highest content in the anthocyanin fraction. The main compound of the copigment fraction was punicalin, a hydrolysable tannin, commonly found in pomegranate juices in high concentrations [34]. Punicalin is an effective natural antioxidant, which showed a higher radical scavenging activity than vitamin C [35]. Moreover, the anthocyanin content correlates with antioxidant potential [36]. Therefore, these compounds can be identified as major contributors inducing the antioxidant and radical scavenging activity observed in the present study.

Similar to the low TPC, the TEAC of pomegranate juice was significantly lower than the extracts. While Seeram et al. [3] and Kozik et al. [29] documented a TEAC value of 416–1912 µmol TE/100 mL, the result of the present study was slightly lower, with 214 µmol TE/100 mL. In the case of the TEAC assay and the ESR spectroscopy analyses using galvinoxyl and DPPH radicals, the XAD-7 extract of pomegranate had the highest radical scavenging activity of all samples. Similar results of radical scavenging were documented by Aloqbi et al. [37] and Les et al. [15], who showed a dose-dependent reduction of free DPPH and H_2_O_2_ radicals induced by pomegranate juice. According to Gil et al. [7], pomegranate juices showed a higher activity than red wine and green tea, which was determined by TEAC, DPPH, and FRAP assay. Moreover, compared to other fruits, the antioxidant potency of juice made from pomegranates is even higher than blueberry and cranberry juice [3], both associated with a protective radical scavenging activity [38]. However, different solvent compositions, extraction techniques, and times should be taken into account.

For the polymeric fraction, its antioxidant potential in the TEAC assay and the TPC were both tendentially lower than the other fractions, which excludes the probability of being the most efficient antioxidant fraction in pomegranates. For this reason, the polymeric fraction was not characterized in more detail or further analyzed by ESR spectroscopy. While the pomegranate XAD-7 extract had the highest radical scavenging activity, its fractions showed no significant differences in reducing galvinoxyl and DPPH radicals. Moreover, a comparable high antioxidant potential determined by the TEAC assay was also detected for the recombined fraction. These results hypothesized that synergistic effects of anthocyanins, copigments, and/or the polymeric fraction including the antioxidant recycling system such as tocopherol and ascorbic acid increased the radical scavenging effect. Such synergistic effects of anthocyanins, flavonol glycoides, and proanthocyanidins of a cranberry extract were already described by Seeram et al. [22]. Therefore, similar effects may be responsible for the higher radical scavenging activity of the XAD-7 extract of the present study. To the best of our knowledge, the galvinoxyl and DPPH radical scavenging activities of the pomegranate fractions have not been analyzed thus far by ESR spectroscopy, whereas for a pomegranate extract obtained by 70% acetone, the scavenging activity against hydroxyl and superoxide radicals has been shown [26]. Moreover, for nine fresh pressed and commercial pomegranate juice samples, obtained from different countries, the DPPH radical scavenging activity using ESR was analyzed [29]. Although the three major anthocyanidins delphinidin, cyanidin, and pelargonidin, as authentic compounds, scavenged the hydroxyl and superoxide radicals, they did not scavenge nitric oxide [26]. As reported in an earlier study [7], the content of hydrolyzable tannins was considered to be predominantly associated with the high radical scavenging activity of pomegranate juices. In contrast to this, the results of the recent study showed no significant difference between the anthocyanin and copigment fraction of pomegranate, thus indicating an equal importance of the anthocyanin fraction.

In the case of the in vitro assays, the ROS-inducing and cytotoxic potential of the used concentrations of PE, as well as the fractions (without any use of H_2_O_2_) was analyzed, whereby no significant effects were documented and could be excluded by the interpretation of the following results. Nevertheless, the non-significant increase in cell viability requires an explanation. The principle of the MTT assay, measuring the mitochondrial dehydrogenase activity, could be influenced by the PE and fractions, leading to the slightly higher cell viability. Wang et al. [39] analyzed the antiproliferative activity of the green tea polyphenol (−)-epigallocatechin-3-gallate on different tumor cell lines by several cytotoxicity assays. The results of the MTT assay showed divergent effects compared to the other assays and detected a higher cell viability after 24 h of incubation [39]. Hence, the PE and fractions of the present study may increase the activity of mitochondrial dehydrogenase, which led to the higher cell viability.

The pomegranate XAD-7 extract as well as the different fractions significantly reduced the cellular oxidative stress in HepG2 cells in the pretreatment experiments for the ROS-reducing activity, whereby the results were nearly similar for all test samples. These antioxidant effects may be correlated to the radical scavenging properties against DPPH and galvinoxyl radicals. Thus, the already discussed results of pomegranate XAD-7 extract in the cell-free system agreed with the antioxidant effects of the in vitro assays, except in terms of possible synergistic effects, which would lead to a higher antioxidant potential for the XAD-7 extract. Nevertheless, the high antioxidant potential of pomegranate extracts has already been described in several in vitro studies [15,40,41]. According to Les and coworkers [15], PE generated from juice partly protected HepG2 cells against oxidative stress and increased the cell survival after treatment with H_2_O_2_ from 60% to 80%. Moreover, H_2_O_2_-induced ROS in keratinocytes were reduced by prior incubation with PE [40]. Similar results were detected by Pontonio et al. [41], who showed a significant scavenging of intracellular H_2_O_2_ radicals by pomegranate juice in murine fibroblasts.

It is well known that H_2_O_2_ and especially ROS can lead to DNA strand breaks [42]. Therefore, the protection against genotoxicity induced by oxidative stress would be interesting in further studies. Forouzanfar and coworkers [43] analyzed the potential of PE inhibiting genotoxicity, although no differentiation between the anthocyanin and copigment fractions was performed. A concentration of 6.25 µg/mL PE showed no significant effects in the Comet assay, whereas 400 µg/mL and 800 µg/mL led to a strong reduction of DNA damage induced by serum/glucose deprivation in PC12 cells [43]. Similar protective effects were also documented for blueberry extracts [44] or an anthocyanin-rich fruit juice, mainly consisting of red grape and blueberry juice [13]. Thus, similar positive results would be expected for the pomegranate XAD-7 extract and especially the anthocyanin fraction. Moreover, in future studies, the bioavailability and the intracellular mechanisms, which were induced by the extract and its fractions after uptake, should be analyzed in more detail. Such results may contribute to the preparation of nutraceuticals on the basis of pomegranate extract.

## 5. Conclusions

Pomegranate and its anthocyanin and copigment fractions contain bioactive phytochemicals that may have beneficial effects on human health. The main anthocyanins in pomegranates are cyanidin-3,5-diglucoside and cyanidin-3-glucoside. Punicalin, a hydrolyzable tannin, was the main compound of the copigment fraction, which represents the main part (74.9%) of the pomegranate XAD-7 extract. All tested samples (pomegranate XAD-7, anthocyanin, and copigment fraction) showed radical scavenging activity against glavinoxyl and DPPH radicals using ESR spectroscopy in a dose-dependent manner. However, pomegranate XAD‑7 extract showed the highest activity, suggesting synergistic effects of anthocyanins, hydrolyzable tannins, and/or polymeric compounds inducing the higher radical scavenging activity. Accordingly, an inhibition against H_2_O_2_-induced oxidative stress was observed in cultured HepG2 cells, whereas the pomegranate XAD-7 extract and the different fractions served as cellular ROS scavengers. In summary, our results suggest free radical scavenging and cellular antioxidant activity of pomegranate XAD-7 extract and its anthocyanin and copigment fraction. Therefore, pomegranate could be used as an ingredient in functional foods and nutraceuticals.

## Figures and Tables

**Table 1:** Identification of the compounds of an anthocyanin and copigment fraction of the XAD-7 extract of pomegranate (*Punica granatum* L.) by HPLC electrospray ionization tandem mass spectrometry (HPLC–ESI–MS/MS).

Peak	[M + H]^±^	Fragments (*m*/*z*)	Compound
A1	627	465, 303	delphinidin-3,5-diglucoside
A2	611	449, 287	cyanidin-3,5-diglucoside
A3	465	303	delphidin-3-glucoside
A4	449	287	cyanidin-3-glucoside
A5	433	271	pelargonidin-3-glucoside
C1	781	601, 271	punicalin
C2	1083	601, 299, 271	punicalagin I
C3	783	601, 301	pedunculagin I
C4	1083	601, 299, 271	punicalagin II
C5	783	601, 301	pedunculagin II
C6	951	907, 301	granatin B
C7	783	601, 301	pedunculagin III
C8	1083	601, 301	pedunculagin III
C9	799	479, 301	ellagic acid derivative
C10	801	347, 301, 195	punicluconin
C11	463	301, 257	ellagic acid hexoside
C12	301	258	ellagic acid
